# Distribution characteristics and driving factors of potentially toxic elements in the soil of *Hippophae rhamnoides* forests in the reclaimed coal - mine areas of ordos

**DOI:** 10.3389/fpls.2026.1947107

**Published:** 2026-10-21

**Authors:** Jiamin Guo, Yan Gao, Guangquan Liu, Hongwu Zhang, Genbatu Ge, Ning Ai, Fangfang Qiang

**Affiliations:** 1 School of Life Sciences, Yan’an University/Key Laboratory of Applied Ecology of Loess Plateau, Shaanxi Provincial Department of Education, Yan’an, Shaanxi, China; 2 National Seabuckthorn Development and Management Center (Plant Development and Management Center for Soil and Water Conservation, Ministry of Water Resources), Beijing, China; 3 China Institute of Water Resources and Hydropower Research, Beijing, China; 4 Yellow River Research Center, Tsinghua University, Beijing, China; 5 College of Geographical Science, Inner Mongolia Normal University, Hohhot, Inner Mongolia, China

**Keywords:** artificial *Hippophae rhamnoides* forest, coal - mine reclamation area, ecological risk, partial least squares structural equation model, soil potentially toxic elements

## Abstract

To investigate the distribution characteristics and driving factors of soil potentially toxic elements after land reclamation in a mining area, this study focused on the soil potentially toxic elements distribution in a Hippophae rhamnoides plantation within the Juxinlong Coal Mine reclamation area in Ordos, Inner Mongolia. Soil sampling gradients were established at distances of 1/8 (0.25 m), 1/4 (0.5 m), and 1/2 (1 m) of the inter-tree distance from the standard tree. Abandoned grassland was used as a control group. The contents and distribution characteristics of potentially toxic elements including Cadmium (Cd), Copper (Cu), Lead (Pb), Chromium (Cr), and Arsenic (As) in the soil were analyzed to assessed their potential ecological risks, and identified the driving factors. The results showed that the contents of Pb, Cu, Cr, Cd, and As in the study area increased as the distance from the tree trunk decreased. The contents of Pb, Cu, Cr, and Cd in the abandoned grassland were generally higher than those in the Hippophae rhamnoides treatment groups, while As showed significant enrichment at the 1/8 plant spacing distance, with its content increasing from 4.03 mg·kg⁻¹ to 9.35 mg·kg⁻¹.The comprehensive potential ecological risk index (RI) increased as the distance decreased, reaching an extremely high-risk level near the standard trees (1/8 plant spacing), with RI values ranging from 434.58 to 581.17, and Cr was identified as the key risk factor. Soil moisture indicators (field water-holding capacity, CWC; saturated water content, SW) were the core driving factors regulating the contents of As, Cd, and Cu, exerting significant negative direct effects (with path coefficients of -0.84, -0.80, and -0.50, respectively). Furthermore, soil moisture indirectly influenced potentially toxic elements distribution by affecting soil pH, available potassium, organic matter, and bacterial community diversity. This study elucidates the distribution pattern of soil potentially toxic elements and their main influencing factors in a coal mine reclamation area, providing a theoretical basis for risk identification and precise management in mining area ecological restoration.

## 1 Introduction

Coal mining areas are typical regions subject to intense anthropogenic disturbance. Long-term mineral resource extraction has led to extensive land degradation and soil potentially toxic elements pollution, drawing continuous attention from scholars globally regarding ecological restoration and risk management (Zhou et al., 2026; Wang et al., 2024).Ordos, as a crucial energy base in China, faces a series of eco-environmental problems from large-scale open-pit mining, including soil degradation, vegetation destruction, and heavy metal accumulation (Liu et al., 2025). To restore land productivity in mining areas and achieve sustainable development, land reclamation has become an inevitable choice. *Hippophae rhamnoides*, known for its drought tolerance, barren resistance, strong nitrogen-fixing ability, and favorable economic benefits, is widely used for vegetation restoration in the Ordos coal mine reclamation area. Studies indicate that the planting of *Hippophae rhamnoides* forests contributes to the improvement and enhancement of soil physicochemical properties, microbial communities, and the micro-domain environment, thereby influencing the occurrence forms and spatial distribution of soil potentially toxic elements (Ren et al., 2025).

As one of the primary pollutants in mining areas, the accumulation level, spatial differentiation, and ecological risk of soil potentially toxic elements are directly related to the safety and stability of reclaimed ecosystems (Sepúlveda Olea et al., 2026). A substantial body of literature has investigated the pollution characteristics, spatial distribution, source apportionment, and ecological risk of soil potentially toxic elements in mining areas, establishing a relatively systematic research framework (Yin et al., 2025; Setu and Strezov, 2025; Zhu et al., 2026; Wang et al., 2025). To identify the key factors influencing heavy metal distribution, studies have frequently employed methods such as principal component analysis, correlation analysis, and multiple regression to pinpoint critical influencers like soil physicochemical properties and microbial communities (Deng et al., 2025; Tang et al., 2025). Furthermore, multivariate statistical methods like Structural Equation Modeling have been used to clarify the direct and indirect driving pathways of soil physical, chemical, and biological factors on potentially toxic elements, revealing the synergistic mechanisms affecting the occurrence state and spatial distribution of potentially toxic elements (Ye et al., 2025; Sun et al., 2024). In research concerning the association between vegetation restoration and potentially toxic elements pollution, it is widely acknowledged that reclaimed vegetation plays a pivotal role in the immobilization and migration regulation of potentially toxic elements in mining areas (Wlodarczyk et al., 2025). Regional empirical studies have demonstrated that soil potentially toxic elements contents in coal mine reclamation areas are significantly higher than those in undisturbed natural areas, exhibiting distinct characteristics of anthropogenic cumulative pollution (Zhang J. et al., 2023). Further investigations into typical mining areas, such as open-pit coal mines at the Shaanxi-Inner Mongolia border and stone coal mining areas in the Zijiang River Basin, have revealed significant spatial heterogeneity in potentially toxic elements pollution characteristics across different sites. In some areas, potentially toxic elements like cadmium pose light to moderate pollution risks, with even severe pollution observed in localized spots, leading to significant variations in pollution loads and ecological risks (Zhu et al., 2026; Dong et al., 2025). In recent years, with the rapid development of phytoremediation technologies, research on the mechanisms of plant-based potentially toxic elements remediation in mining areas has deepened. Abhishek et al. systematically reviewed the phytoremediation technology systems for mine solid waste accumulation areas, such as fly ash and red mud zones. They clarified the remediation mechanisms of potentially toxic elements-tolerant plants and optimization pathways for biotechnology, confirming that vegetation restoration is a green and efficient approach for the *in-situ* immobilization and pollution control of potentially toxic elements in mining areas (Abhishek et al., 2025a). Building on this, Abhishek et al. further screened various potentially toxic elements-tolerant herbaceous plants and elucidated the stabilization remediation mechanisms of dominant plants, providing theoretical support for the selection of plant species for vegetation restoration in mining areas (Abhishek et al., 2025b). Meanwhile, Chakraborty et al. precisely analyzed the source differences of potentially toxic elements in coal mining areas and their human health risks based on multi-variate coupling models. This work refined the risk assessment system for potentially toxic elements pollution in mining areas, offering new research perspectives for the precise management of potentially toxic elements (Chakraborty et al., 2026).

Although current research has basically clarified the pollution characteristics, spatial distribution patterns, and basic risk levels of soil potentially toxic elements in mining areas, and has also initially revealed the regulatory effects of factors such as the soil environment, vegetation, and microorganisms on the distribution of potentially toxic elements. Moreover, recent research has gradually focused on the mechanisms of phytoremediation and refined risk assessment, further improvement is needed in the analysis of the distribution characteristics and driving factors of soil potentially toxic elements in reclaimed areas, centered on single - plant vegetation and in combination with soil physical and chemical properties and microbial community characteristics. In particular, there is a lack of systematic explanations on how soil physical and chemical properties, microbial functions, and vegetation characteristics regulate the migration and fixation processes of potentially toxic elements.

Therefore, this study focuses on soil potentially toxic elements in the artificial *Hippophae rhamnoides* forests formed after the reclamation of the Juxinlong Coal Mine in Ordos, Inner Mongolia. By establishing sampling gradients at different distances from standard trees (1/8, 1/4, and 1/2 of the plant spacing) and using unreclaimed abandoned grassland as a control, the following studies are systematically conducted: (1) to reveal the spatial distribution characteristics of Cd, Cu, Pb, Cr, and As in the soil; (2) to assess the potential ecological risk levels of soil in the reclaimed area; and (3) to construct a partial least squares structural equation model (PLS-SEM) to analyze the direct and indirect driving pathways of soil physical, chemical, microbial, and vegetation factors on potentially toxic elements distribution. This study aims to uncover the spatial distribution patterns of soil potentially toxic elements and their underlying driving factors after vegetation restoration in coal mine reclamation areas, providing a scientific basis for risk identification and regulation of soil potentially toxic elements in ecological restoration of mining areas.

## 2 Materials and methods

### 2.1 Study area

The study area is located in the Juxinlong Coal Mine (110°4’2”E, 39°54’16”N) in the eastern part of Dongsheng District, Ordos City, Inner Mongolia Autonomous Region. The altitude is approximately 1360 m, the terrain is flat, and water and wind erosion are severe. This region is characterised by a temperate continental monsoon climate with a frost-free period of about 135 days. Annual precipitation varies greatly from year to year, averaging around 190–400 mm, mainly concentrated from July to September. Since 2011, ecological restoration of the coal mining wasteland in the study area has been carried out in collaboration with the Gaoyuan Shengguo Seabuckthorn Products Co., Ltd. The main tree species include Chinese seabuckthorn (*Hippophae rhamnoides subsp. sinensis*), large-fruited seabuckthorn (*Hippophae rhamnoides subsp. gyantsensis)*, and tamarisk (*Tamarix chinensis Lour.*). Artificial grass seeding primarily uses *Achnatherum splendens(Trin)Nevski* The location map and plot photos of the study area are shown in **Figure 1**.

**Figure 1:**
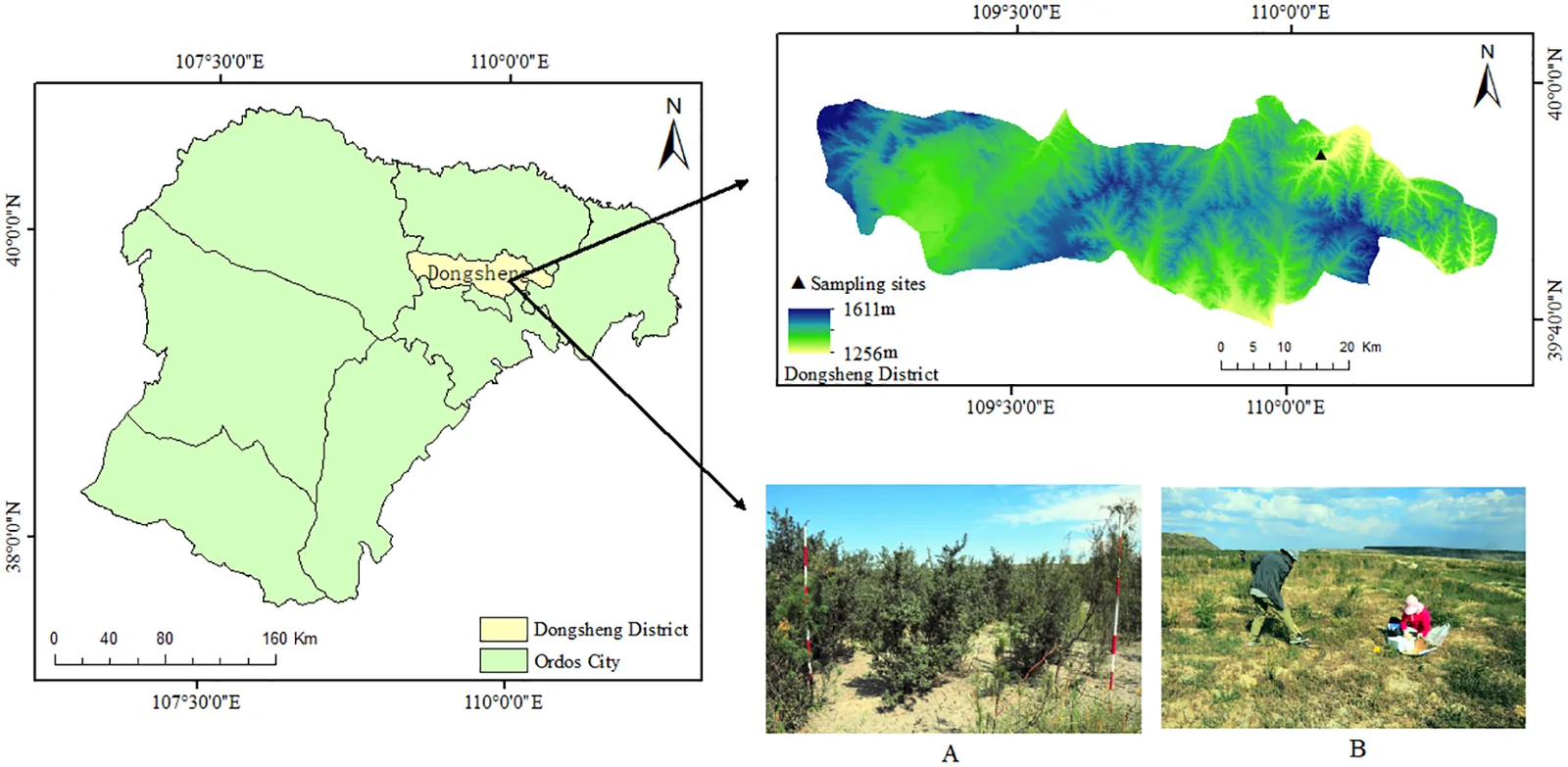
Location of the study area and sampling plots. The inset in the upper left shows the geographic location of the study area in China. The upper-right panel is the digital elevation model (DEM) of Dongsheng District, with elevation in meters (m). **(A)** represents artificial *Hippophae rhamnoides* forest (E: 110°4’2”–110°4’45”, N: 39°53’53”–39°54’27”), **(B)** represents abandoned land.

### 2.2 Soil sample collection and processing

Through comprehensive surveys and investigations of the reclaimed area of Juxinlong Coal Mine in Ordos, typical artificial *Hippophae rhamnoides* forests formed after ecological restoration were selected as the research objects. Five standard plots of 20 m × 20 m were established for sampling, with unreclaimed abandoned grasslands surrounding the reclaimed area serving as controls. A complete survey of *Hippophae rhamnoide*s and understory vegetation in each standard plot was conducted using the individual-tree measurement method to calculate the standard trees for each plot. Three *Hippophae rhamnoides* plants similar to the standard trees were selected within each plot for sampling and analysis. Centered on the standard *Hippophae rhamnoides* tree, soil sampling points were selected at distances of 0.25 m, 0.5 m, and 1 m from the root (corresponding to 1/8, 1/4, and 1/2 of the plant spacing in the *Hippophae rhamnoides* forest, as shown in **Figure 2**). Soil profiles of 20 cm depth were excavated for layered sampling (0–10 cm and 10–20 cm), with three replicates per layer. Fresh soil samples were divided into two parts. One part was stored at -80 °C for total DNA extraction and subsequent high-throughput sequencing to assess bacterial community diversity (Chao1, Shannon, and Simpson indices) and structure. The other part was air-dried, ground, and sieved for the determination of soil physicochemical properties and potentially toxic elements. A total of 288 soil samples were collected. The specific composition was calculated as follows: 5 (standard plots) × 3 (standard trees/profiles) × 3 (distances from standard trees) × 2 (soil layers) × 3 (replicates) + 1 (abandoned grassland) × 3 (profiles) × 2 (soil layers) × 3 (replicates) = 288 samples. The relevant soil physicochemical properties and microbial community compositions are shown in **Table 1** and **Figure 3**.

**Figure 2:**
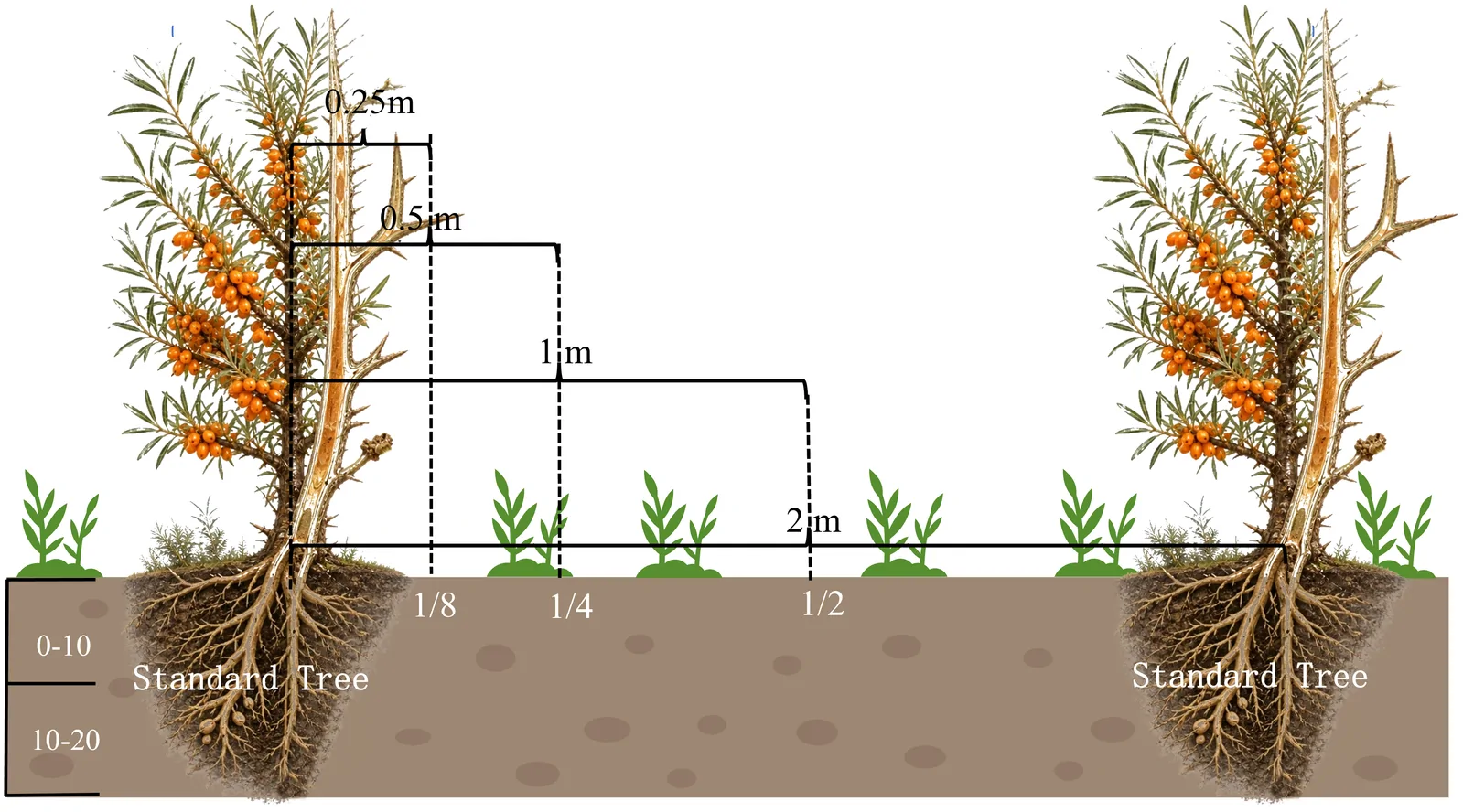
Schematic diagram of distances from the standard *Hippophae rhamnoides* tree.

**Table 1:** Variations in soil physicochemical properties at different distances from standard tree. Note: Data are presented as mean ± standard deviation.

Indicator	1/2	1/4	1/8	CK
pH	8.08 ± 0.419	8.05 ± 0.41	8.09 ± 0.47	7.82 ± 0.03
EC/mS·cm^-^¹	125.68 ± 28.77	127.85 ± 26.91	135.44 ± 25.46	143.01 ± 15.9
AN/mg·kg^-^¹	9.08 ± 2.33	9.46 ± 2.48	9.58 ± 2.23	3.45 ± 1.44
TP/g·kg^-^¹	0.53 ± 1.12	0.53 ± 0.18	0.53 ± 0.14	0.50 ± 0.20
AK/mg·kg^-^¹	139.60 ± 43.88	130.08 ± 43.97	116.44 ± 44.09	42.93 ± 5.92
AP/mg·kg^-^¹	21.06 ± 13.45	18.43 ± 10.08	17.53 ± 12.45	24.46 ± 0.82
SOM/g·kg^-^¹	5.85 ± 3.22	4.94 ± 3.00	4.77 ± 2.63	9.28 ± 3.21
SWC/%	41.36 ± 9.29	37.41 ± 5.62	41.46 ± 5.17	36.44 ± 4.39
NCP/%	12.91 ± 4.11	10.75 ± 4.00	14.15 ± 2.77	7.73 ± 2.39
CP/%	35.15 ± 6.30	35.95 ± 3.16	33.3 ± 3.53	36.52 ± 4.84
CWC/%	31.41 ± 7.16	29.12 ± 3.42	31.02 ± 5.54	30.02 ± 4.27
BD/g·cm^-^³	1.19 ± 0.14	1.27 ± 0.14	1.15 ± 0.10	1.22 ± 0.07
Tpor/%	48.07 ± 6.56	46.70 ± 2.88	47.45 ± 3.74	44.26 ± 3.86
SW/%	7.08 ± 2.66	7.09 ± 1.96	7.05 ± 2.77	10.71 ± 1.48
Clay/%	3.17 ± 0.87	3.49 ± 0.91	3.29 ± 0.85	3.70 ± 0.31
Silt/%	56.36 ± 11.61	58.53 ± 11.26	58.14 ± 11.28	68.44 ± 2.02
Sand/%	40.46 ± 12.30	37.98 ± 12.00	38.56 ± 11.93	27.85 ± 2.05

**Figure 3:**
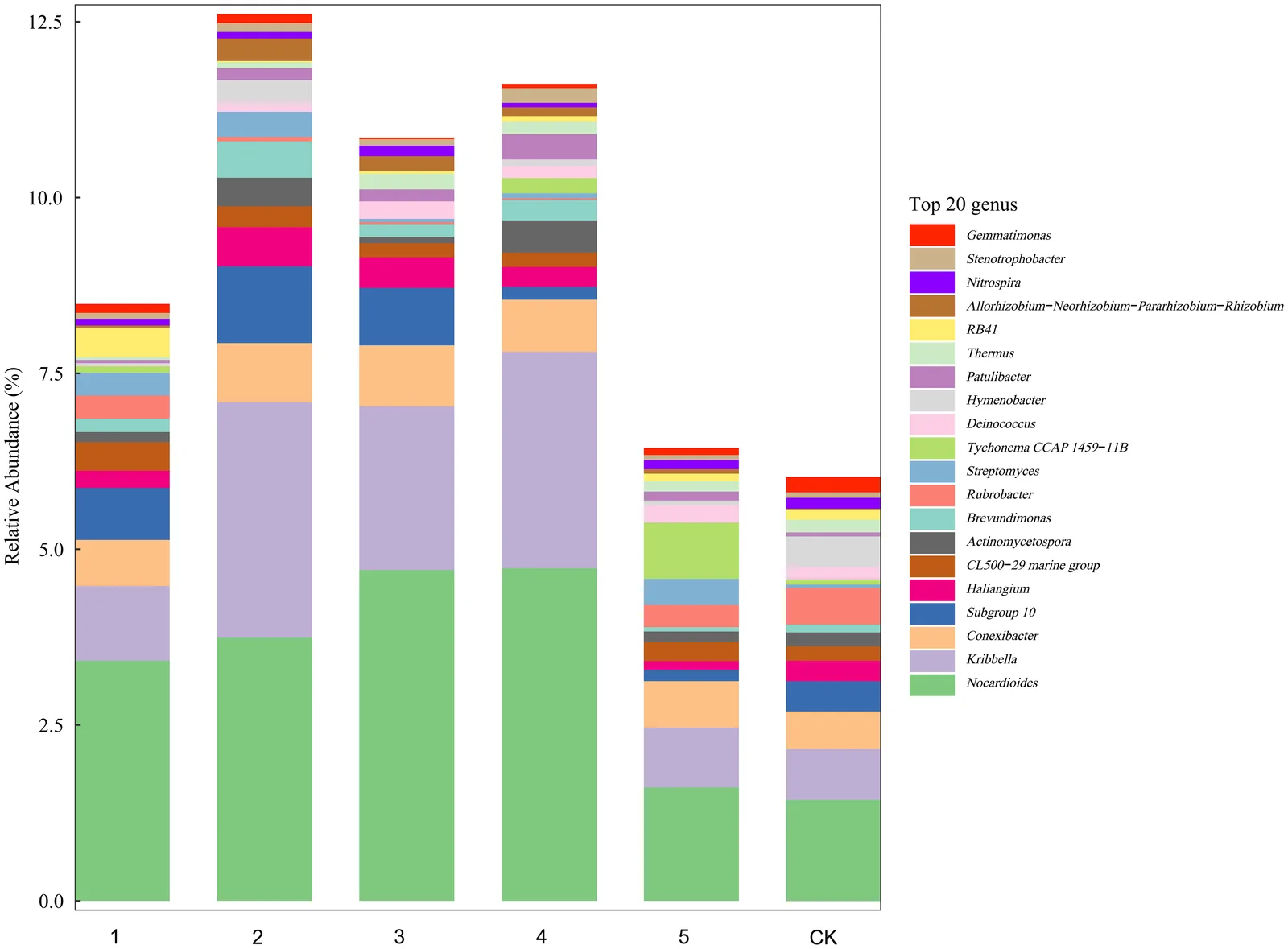
Structural characteristics of soil bacterial community composition in *Hippophae rhamnoides* forestland.

Meanwhile, five 1 m × 1 m herbaceous quadrats were selected in each standard plot to investigate the characteristics of the herbaceous community, such as species composition, plant height, and coverage. A total of 30 herbaceous quadrats were surveyed, calculated as: 6 (5 *Hippophae rhamnoides* forest standard plots + 1 abandoned grassland) × 5 (replicates) = 30 quadrats.

### 2.3 Measurement indicators and methods

Soil water content (SWC) was determined using the oven-drying method. Soil bulk density (BD), total porosity (Tpor), saturated water content (SW), capillary water holding capacity (CWC), capillary porosity (CP), and non-capillary porosity (NCP) were measured and calculated using the ring knife immersion method. Soil particle size was measured using a BT-9300S laser particle size analyzer (Liaoning, China), with soil particle size classification based on the US soil texture classification standard. Soil pH and electrical conductivity (EC) were measured using an HS-320 high-precision intelligent acidity meter (Chengdu, China) and a DDS-608 multifunctional conductivity meter (Chengdu, China), respectively, with a soil-to-water ratio of 1:2.5. Available nitrogen (AN) was quantified using the alkaline hydrolysis-diffusion method. Available potassium (AK) was determined using the ammonium acetate extraction-flame photometry method. Available phosphorus (AP) was measured using the 0.5 mol·L^-^¹ sodium bicarbonate method. Total phosphorus (TP) was determined using the sodium hydroxide fusion method. Soil organic matter (SOM) was determined using the potassium dichromate volumetric method. A 0.5 g subsample of soil passing through a 100-mesh sieve was accurately weighed for microwave digestion. After digestion in a microwave digestion and extraction instrument, the contents of Cd, Cu, Pb, and Cr in the digest were determined using an atomic absorption spectrophotometer, while the content of As was determined using an atomic fluorescence spectrophotometer. The quality and precision of the measurements were controlled using the certified reference material GSB07-3272-2015.

A 0.5 g subsample of the fresh mixed soil sample was taken, and the Omega Soil DNA Kit (Omega Bio - tek, USA) was used to extract the total soil DNA. Then, conduct quality verification through 1% agarose gel electrophoresis and a NanoDrop 2000 spectrophotometer (Thermo Scientific, USA). The DNA concentration should be > 20 ng/μL, and the A260/A280 ratio should be between 1.8 and 2.0. For the analysis of the bacterial community, select the 341F/805R primers to perform PCR amplification on the V3 - V4 hypervariable regions of the 16S rRNA gene. The amplified products are subjected to paired - end sequencing on the Illumina NovaSeq 6000 platform, with an average of 45,000 - 60,000 effective sequences per sample obtained from the bacterial library. After the amplified products were purified by nucleic acid purification magnetic beads, a fluorescent quantifier is used for quantitative analysis. The sequencing data are subjected to quality control and denoising through the DADA2 pipeline in QIIME2, and clustered into amplicon sequence variants (ASV) at a 99% similarity level. Based on the ASV sequences, use the SILVA database (v138) to perform taxonomic annotation of the bacteria, and calculate indicators such as the Chao1, Shannon, and Simpson diversity indices (Reyes-Ardila et al., 2024).

### 2.4 Data processing and analysis

#### 2.4.1 Potential ecological risk index (PERI)

The individual potential ecological risk index (PERI,Equation 1) and the comprehensive potential ecological risk index (RI,Equation 2) were calculated according to the following equations:

(1) $$PERI=C_{f}\cdot T_{r}^{i}$$

(2) $$RI=\sum_{i=1}^{n}PERI$$

Where: C_f_ is the measured content of single element *i* in the soil sample (mg·kg^-^¹); $T_{r}^{i}$ is the biological toxicity factor forelement *i* (As=10, Cd=30, Cr=2, Cu=5, Pb=5). The grading criteria for PERI and RI (Hakanson, 1980; Xie et al., 2026) are shown in **Table 2**.

**Table 2:** Classification criteria for single potential ecological risk index and comprehensive potential ecological risk index.

Pollution level	Slight risk	Moderate risk	Strong risk	Very strong risk	Extremely strong risk
PERI	PERI ≤ 40	40<PERI ≤ 80	80<PERI ≤ 160	160<PERI ≤ 320	PERI>320
RI	RI ≤ 110	110<RI ≤ 220	220<RI ≤ 440	—	RI>440

#### 2.4.2 Statistical analysis

Microsoft Excel 2021 was used for systematic data organization and preprocessing of basic data including soil physical properties, vegetation community composition, and potentially toxic elements contents, One-way analysis of variance (ANOVA) was used to compare the soil potentially toxic elements contents at different distances from the standard tree and their differences with the abandoned grassland control (CK) Subsequently, statistical analysis was performed using R language (version 4.5.2). The spatial distribution characteristics of soil potentially toxic elements were plotted using the ggplot2 package. The plspm package was used to construct a Partial Least Squares Structural Equation Model. In this model, soil moisture was represented by field water-holding capacity (CWC) and soil water content (SWC); soil chemical properties were indicated by pH, available potassium (AK), and soil organic matter (SOM); soil bacterial community was characterized by Chao1, Simpson, and Shannon indices; and soil potentially toxic elements contents were measured by the actual concentrations of Cd, Cu, Pb, Cr, and As. The model fit was assessed using R2 and the Goodness-of-Fit (GoF) index. Additionally, trend charts of soil potentially toxic elements changes along different gradients were plotted using Origin 2024.

## 3 Results

### 3.1 Changes in the content of soil potentially toxic elements

As shown in **Figure 4** this study analyzed the variation characteristics of soil potentially toxic elements (Pb, Cu, Cr, Cd, and As) contents at different distances from the standard tree (1/2, 1/4, and 1/8 of the plant spacing), using abandoned grassland (CK) as a control. Overall, tree planting influenced the enrichment of potentially toxic elements in the surrounding soil, resulting in differences in potentially toxic elements contents across different distance gradients. Specifically, the contents of Pb, Cu, Cr, Cd, and As generally showed an increasing trend as the distance from the standard tree decreased (from 1/2 to 1/8 of the plant spacing). Notably, the As content at 1/8 of the plant spacing increased from 4.03 mg·kg^-^¹ to 9.35 mg·kg^-^¹, demonstrating significant enrichment characteristics (p<0.05). Compared with the treatment groups, the CK group exhibited the highest median contents of Pb (15.94 mg·kg^-^¹) and Cr (142.87 mg·kg^-^¹), as well as significantly higher contents of Cu and Cd than those in any of the treatment groups. In contrast, As showed an opposite pattern, with significantly higher contents in the treatment groups than in the CK group.

**Figure 4:**
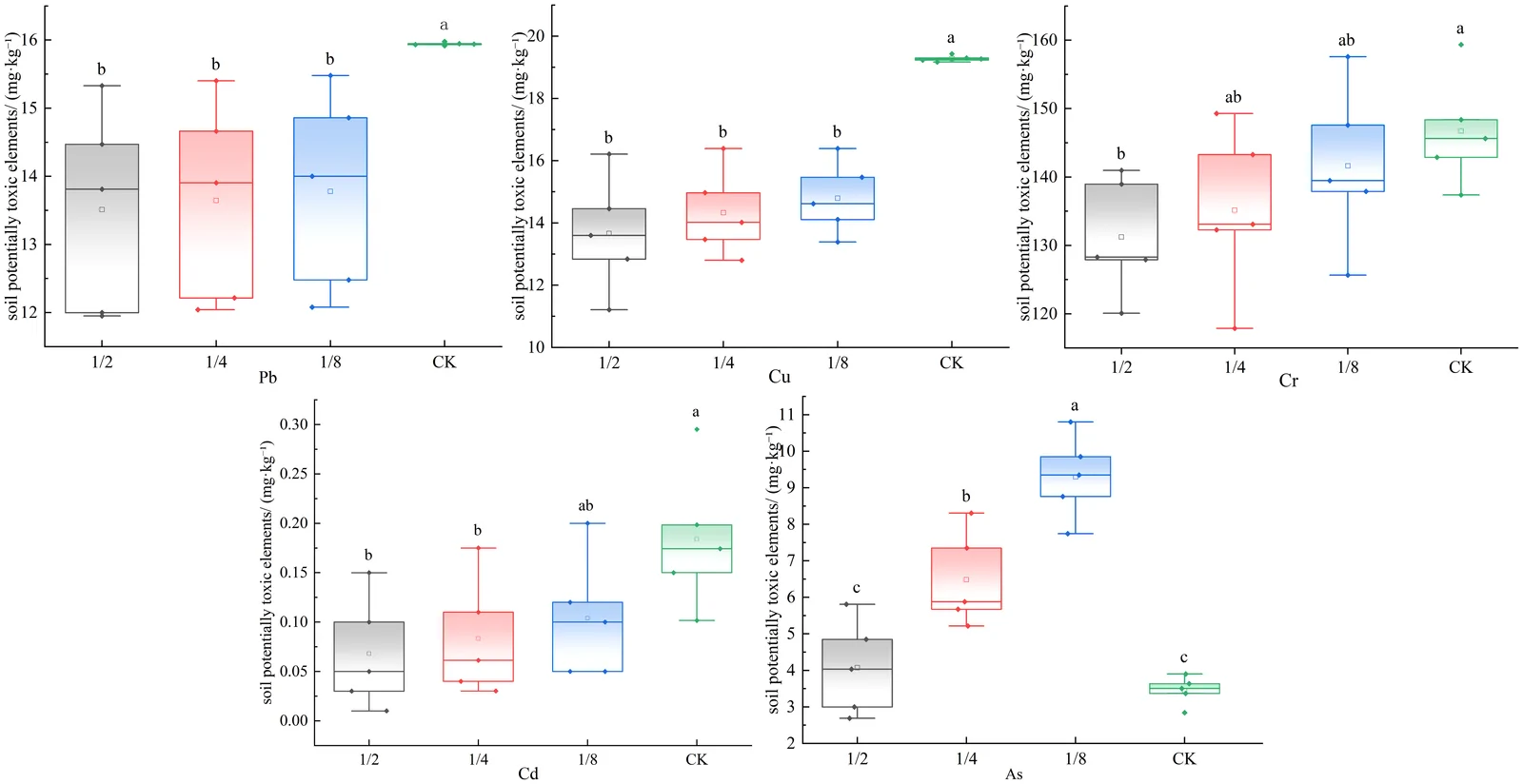
Changes in soil potentially toxic elements under different standard tree distance. Different lowercase letters indicate significant differences among treatments at p<0.05, individual data points are shown as scatter dots.

### 3.2 Evaluation of soil pollution index

According to the data in **Table 3** and the classification criteria in **Table 2** the average RI values for all distances from the standard tree and the CK control group ranged from 432.44 to 533.03, indicating an extremely high risk level, with the highest RI value observed at 1/8 of the plant spacing. The average PERI values for Cr at all distances (258.56–318.72) were at a very high risk level, making it the primary contributor to ecological risk. The average PERI values for As (40.04–93.01) increased from moderate to high risk as the distance decreased. The average PERI values for Cu and Pb ranged from moderate to high risk levels, while Cd consistently remained at a slight risk level. Overall, the comprehensive potential ecological risk of soil potentially toxic elements showed an increasing trend as the distance from the standard tree decreased (from 1/2 to 1/8 of the plant spacing), with Cr identified as the key risk factor.

**Table 3:** PERI and RI statistics of potential toxic elements in soil at different standard tree distances.

Distance	Indicator	As	Cd	Cr	Cu	Pb	RI
1/2	maximum	58.11	4.50	281.94	81.07	76.64	494.67
	minimum	26.90	0.30	220.15	26.04	59.76	333.15
	average	40.04	1.85	258.56	63.47	68.52	432.44
1/4	maximum	83.06	5.25	298.56	81.96	77.01	538.36
	minimum	52.16	0.91	235.73	33.99	60.21	383.86
	average	64.85	2.50	270.32	65.65	68.23	471.55
1/8	maximum	108.01	6.00	315.18	81.96	77.39	581.17
	minimum	77.43	1.50	251.30	41.94	60.42	434.58
	average	93.01	3.14	283.28	68.98	68.90	517.31
CK	maximum	39.03	8.86	318.72	97.15	79.90	533.03
	minimum	28.40	3.05	274.77	95.84	79.58	492.27
	average	34.51	5.52	293.45	96.40	79.72	509.59

### 3.3 Driving factors of the spatial distribution of soil potentially toxic elements

The partial least squares structural equation model (PLS-SEM) was used to analyze the relationship between the content of potentially toxic elements in the soil near the standard tree and soil physicochemical properties, biological indicators, and bacterial communities (**Figure 5**). Soil moisture indicators (CWC and SW) were significantly negatively associated with the contents of As, Cd, and Cu (with path coefficients of -0.84**, -0.82**, and -0.50**, respectively). Meanwhile, they exhibited complex interrelationships with chemical indicators such as soil available potassium (AK), soil organic matter (SOM), and pH, as well as microbial community structure (Chao1, Simpson, and Shannon indices) and biomass. These associative interactions collectively influenced the content of soil potentially toxic elements.

**Figure 5:**
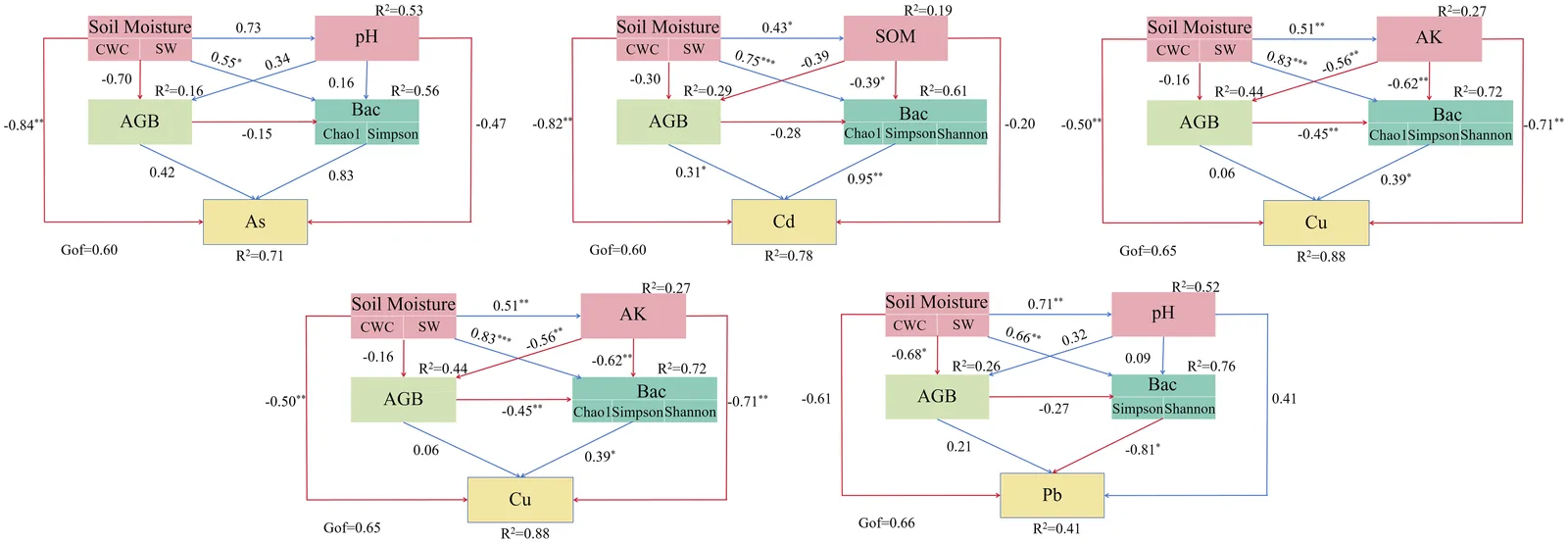
Driving factors of potentially toxic elements in near standard tree. The red arrow indicates negative impact, while the blue arrow indicates positive impact. The number next to the arrow represents the path coefficient, indicating the degree of influence of correlation. ***Indicates significant correlation at the 0.001 level, **indicates significant correlation at the 0.01 level, and *indicates significant correlation at the 0.05 level. Soil Moisture, soil moisture index; CWC, soil capillary water holding capacity; SW, saturated water content; SOM, soil organic matter; AK, available potassium; AGB, aboveground biomass; Bac, bacterial community diversity indices (Chao1, Simpson, Shannon indices).

## 4 Discussion

### 4.1 Distribution characteristics of soil potentially toxic elements at different distances

In this study, sampling gradients were established centered on the standard *Hippophae rhamnoides* trees. The results showed that the contents of Pb, Cu, Cr, Cd, and As were highest at the location closest to the tree trunk (1/8 of the plant spacing, 0.25 m). This phenomenon may be related to the root activity and canopy structure of *Hippophae rhamnoides*. Previous studies have indicated that root exudates of woody plants can alter the pH and organic matter content of rhizosphere soil, promoting the accumulation of potentially toxic elements bound to iron-manganese oxides and organic matter in the rhizosphere (Nong et al., 2023; Podar and Maathuis, 2022; He et al., 2026). Meanwhile, the canopy of *Hippophae rhamnoides* intercepts potentially toxic elements particles from atmospheric deposition, which then accumulate in the soil around the tree trunk after being leached by precipitation (Fang et al., 2024; Meng et al., 2024; Yuan et al., 2024).

Compared with the treatment groups, the contents of Pb, Cu, Cr, and Cd in the abandoned grassland (CK) were higher than those in each treatment group, whereas As exhibited the opposite pattern, being significantly higher in the treatment groups than in CK. This inter-element difference may originate from differences in land use processes and vegetation restoration conditions (Song et al., 2023); The abandoned grassland, having undergone no engineering disturbance or plant improvement, retained a portion of the potentially toxic elements left over from the coal-mining period, whereas during the reclamation of the *Hippophae rhamnoides* forest, soil tillage, backfilling with foreign soil, and plant growth may have diluted or immobilized soil Potentially Toxic Elements to some extent (Dong et al., 2024; Di et al., 2024). In addition, as a pioneer tree species, *Hippophae rhamnoides* possesses a strong root absorption and retention capacity, and may remove part of the Pb, Cu, Cr, and Cd from the soil through phytoextraction, thereby reducing their contents in the understory soil (Dong et al., 2024). Because the abandoned grassland lacked such biological output pathways, Potentially toxic elements tend to accumulate and persist in the surface layer. Meanwhile, the litter layer and improved soil structure of the *Hippophae rhamnoides* forest enhanced the adsorption and immobilization of soil potentially toxic elements by soil organic matter, reducing their mobility (Zhang et al., 2026); by contrast, the bare surface of the abandoned grassland, being subject to wind and water erosion, may have led to relative enrichment of potentially toxic elements carried by fine particles in the surface layer, further intensifying this difference (Qin et al., 2023). The opposite trend of As is related to the metalloid geochemical properties of arsenic. Root activity, rhizosphere organic matter accumulation, and litter return in the trees promoted the adsorption and enrichment of As in the soil near the tree (Budzyńska et al., 2025); meanwhile, plant uptake–aboveground translocation–litter return may also have intensified this spatial difference (Chen et al., 2026). In contrast, the abandoned grassland lacks obvious rhizosphere enrichment and organic matter input, resulting in a lower As content. It should be noted that this study only observed the above spatial distribution patterns and did not directly measure root exudates, potentially toxic elements speciation, or atmospheric deposition fluxes; therefore, the above mechanisms are only possible explanations proposed on the basis of previous studies, and their relative contributions still need to be further verified through rhizosphere process simulation, speciation fractionation, and deposition monitoring.

### 4.2 Driving factors and regulation pathways of soil potentially toxic elements distribution

In this study, the PLS-SEM model was employed to identify the key influencing factors governing the distribution of potentially toxic elements in the soil of the study area. The analysis results indicated that the goodness-of-fit (Gof) of the model ranged from 0.60 to 0.66, demonstrating a robust explanatory power for the relationships among variables.

Soil moisture indicators were significantly negatively correlated with the contents of As, Cd, and Cu, suggesting that moisture conditions inhibit the enrichment of these three potentially toxic elements. A plausible explanation is that increased moisture causes soil particles to disperse, thereby reducing the binding efficiency of potentially toxic elements with soil colloids (Gong et al., 2025; Liu et al., 2024). Simultaneously, changes in moisture can alter the soil redox state, creating anaerobic conditions in relatively moist micro-domains. This promotes the reductive dissolution of iron-manganese oxide minerals, releasing adsorbed As, Cd, and Cu into the soil solution, where they may subsequently migrate or be leached with water flow (Zhang Q. et al., 2023; Chen et al., 2026; Yuan et al., 2026; Luo et al., 2020). This inference aligns with the bacterial community composition characteristics observed in this study (**Figure 3**): among the top 20 bacterial orders in relative abundance, *Betaproteobacteriales*, *Sphingomonadales*, *Myxococcales*, and *Blastocatellales* are groups with potential iron-reducing metabolic capabilities. Their presence may accelerate the reductive dissolution of iron-manganese oxides, thereby facilitating the release and redistribution of As, Cd, and Cu.

The model revealed that soil moisture indicators had indirect effects on Pb, As, Cr, and Cu through mediating variables such as pH or available potassium (AK), with effect values of approximately 0.35, 0.25, 0.1, and -0.1, respectively. Specifically, the positive regulation of pH by moisture may alter the adsorption-desorption equilibrium of potentially toxic elements: under alkaline conditions, Pb, As, and Cr tend to co-precipitate with carbonates or hydroxides, thereby reducing their activity (Li et al., 2021). Meanwhile, the increase in K^+^ concentration resulting from higher soil moisture alters the ion balance on the colloid surface, promoting the desorption of Cr and Cu into the soil solution and facilitating their migration (Dhaliwal et al., 2025; Mu et al., 2023). These chemical processes, superimposed on direct effects, collectively determine the accumulation intensity of potentially toxic elements in the area near the standard trees. Soil moisture indicators indirectly influenced the distribution of potentially toxic elements through the microbial community pathway. Bacterial community composition analysis showed that among the top 20 bacterial orders, *Betaproteobacteriales*, *Sphingomonadales*, *Myxococcales*, and *Blastocatellales* possess potential iron-reducing metabolic capabilities, while *Propionibacteriales*, Actinobacteria groups (*Micrococcales*, *Solirubrobacterales*), *Sphingomonadales*, and *Acidobacteria Blastocatellales* are potential acid-producing microorganisms. These microbes can secrete organic acids to promote mineral weathering or dissolve iron oxides through iron reduction. Changes in moisture can alter soil aeration and redox conditions, thereby affecting the activity and community structure of these functional microorganisms, which in turn influences the speciation and spatial distribution of potentially toxic elements in the soil (Huang et al., 2026; Long et al., 2022). Above-ground biomass (AGB) exerted negative indirect effects on Cd, Cr, and Cu mediated by bacterial diversity, with effect values of -0.2, -0.1, and -0.1, respectively. This indicates that higher AGB is associated with lower potentially toxic elements mediated by bacterial diversity. An increase in AGB typically implies greater litter input and root exudation, which in turn promotes higher soil microbial diversity (Jiao et al., 2025). It should be noted that the aforementioned mechanisms remain speculative explanations based on community composition data and existing literature.

## 5 Conclusion

This study systematically analyzed the spatial distribution characteristics and driving factors of potentially toxic elements in the soil of artificial *Hippophae rhamnoides* forests in the reclaimed coal mine area of Ordos at different distance gradients. The five potentially toxic elements (Pb, Cu, Cr, Cd, and As) under the *Hippophae rhamnoides* forests all showed an enrichment trend of increasing content as the distance from the standard tree decreased. Compared with the abandoned grassland, the *Hippophae rhamnoides* forests significantly reduced the overall contents of Pb, Cu, Cr, and Cd, but led to the local accumulation of As. The comprehensive potential ecological risk index (RI) reached 434.58–581.17 near the standard tree, entering the extremely high-risk level. Among them, the individual potential ecological risk index (PERI) of Cr was at a very high-risk level at all sampling points, making it the main contributing factor to the risk. Soil moisture indicators (field water-holding capacity, CWC; soil water content, SW) were the core driving factors regulating the contents of As, Cd, and Cu, having significant negative direct effects on them, with path coefficients of −0.84, −0.80, and −0.50, respectively. Meanwhile, soil moisture also indirectly affected the distribution of potentially toxic elements through mediating pathways such as altering soil pH, available potassium, organic matter content, and bacterial community diversity. Therefore, in the ecological restoration management of mining areas, attention should be paid to the spatial heterogeneity of risks caused by the enrichment of potentially toxic elements in the rhizosphere area. Differentiated and precise monitoring and control strategies should be implemented to improve the effectiveness of restoration, thereby providing a scientific basis for soil pollution governance and ecological risk control in coal mine reclamation areas.

## Data Availability

Data available on request from the corresponding authors.
